# Ivabradine acutely improves cardiac Ca handling and function in a rat model of Duchenne muscular dystrophy

**DOI:** 10.14814/phy2.15664

**Published:** 2023-04-09

**Authors:** Petra Lujza Szabo, Jessica Marksteiner, Janine Ebner, Christopher Dostal, Bruno K. Podesser, Jakob Sauer, Helmut Kubista, Hannes Todt, Benjamin Hackl, Xaver Koenig, Attila Kiss, Karlheinz Hilber

**Affiliations:** ^1^ Ludwig Boltzmann Institute for Cardiovascular Research at the Center for Biomedical Research and Translational Surgery Medical University of Vienna Vienna 1090 Austria; ^2^ Department of Neurophysiology and Neuropharmacology, Center for Physiology and Pharmacology Medical University of Vienna 1090 Vienna Austria

## Abstract

The muscular dystrophies caused by dystrophin deficiency, the so‐called dystrophinopathies, are associated with impaired cardiac contractility and arrhythmias, which considerably contribute to disease morbidity and mortality. Impaired Ca handling in ventricular cardiomyocytes has been identified as a causative factor for complications in the dystrophic heart, and restoration of normal Ca handling in myocytes has emerged as a promising new therapeutic strategy. In the present study, we explored the hypothesis that ivabradine, a drug clinically approved for the treatment of heart failure and stable angina pectoris, improves Ca handling in dystrophic cardiomyocytes and thereby enhances contractile performance in the dystrophic heart. Therefore, ventricular cardiomyocytes were isolated from the hearts of adult dystrophin‐deficient DMD^mdx^ rats, and the effects of acutely applied ivabradine on intracellular Ca transients were tested. In addition, the drug's acute impact on cardiac function in DMD^mdx^ rats was assessed by transthoracic echocardiography. We found that administration of ivabradine to DMD^mdx^ rats significantly improved cardiac function. Moreover, the amplitude of electrically induced intracellular Ca transients in ventricular cardiomyocytes isolated from DMD^mdx^ rats was increased by the drug. We conclude that ivabradine enhances Ca release from the sarcoplasmic reticulum in dystrophic cardiomyocytes and thereby improves contractile performance in the dystrophic heart.

## Graphical abstract

We have investigated the acute effects of the heart rate‐ lowering drug ivabradine on cardiac Ca handling and function in a rat model of Duchenne muscular dystrophy. Our findings suggest that the drug improves Ca handling in dystrophic cardiomyocytes and thereby enhances contractile performance in the dystrophic heart.

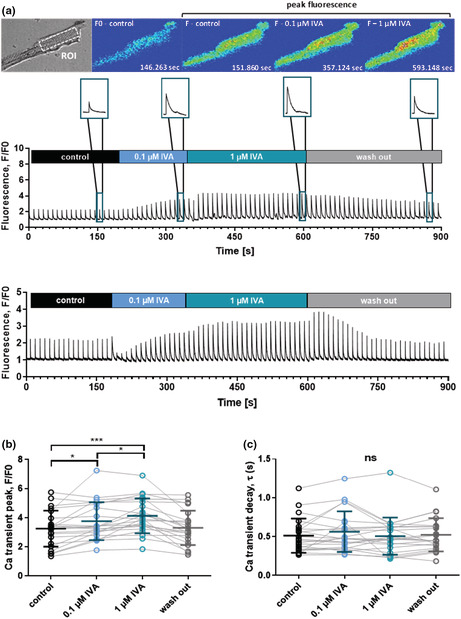

## 1 INTRODUCTION

Ivabradine is a heart rate‐lowering drug clinically approved for the treatment of heart failure and stable angina pectoris (Koruth et al., 2017; Rushworth et al., 2011; Scicchitano et al., 2014). The drug's negative chronotropic effect is thought to rely on the inhibition of hyperpolarization‐activated cyclic nucleotide‐gated (HCN) channels by low micromolar concentrations (IC_50_ values for HCN channel block in vitro: 1–5 μM (Postea & Biel, 2011; Stieber et al., 2006)). Besides its negative chronotropic action, ivabradine also decreases cardiac ventricular conduction velocity (Amstetter et al., 2021), inhibits various cardiac ion channels (Hackl et al., 2022; Haechl et al., 2019), and affects the contractility of normal (Xie et al., 2020) and diseased (Couvreur et al., 2010; Mackiewicz et al., 2014; Ma̧czewski & Mackiewicz, 2008; Navaratnarajah et al., 2013; Reil et al., 2013) hearts. Ivabradine's effects on cardiac contractility are possibly generated via modulation of Ca handling in cardiomyocytes. Thus, in healthy rats, administration of ivabradine had a positive inotropic effect and significantly increased the amplitude of intracellular Ca transients in isolated ventricular cardiomyocytes (Xie et al., 2020).

Only a very limited number of studies have so far considered the potential of ivabradine in the therapy of cardiomyopathies, which occur in the course of the muscular dystrophies. Beneficial effects of ivabradine were reported for the case of a 22‐year‐old patient with Becker muscular dystrophy, where the drug acutely normalized sinus tachycardia and resolved heart failure, presumably by generating a positive inotropic effect (Finsterer et al., 2012). Beneficial effects of long‐term therapy (7‐year follow‐up) with ivabradine in addition to conventional therapy in a Duchenne muscular dystrophy (DMD) patient were reported by De Benedittis et al. These authors encouraged the use of ivabradine in order to improve left ventricular (LV) function in DMD patients (De Benedittis et al., 2016). In line with De Benedittis et al., a recently performed clinical trial, studying the effects of ivabradine on long‐term outcomes in end‐stage DMD cardiomyopathy, suggested a protective role of the drug: the incidence of acute adverse events was reduced, and LV function was improved (Adorisio et al., 2019). Taken together, ivabradine seems to exert beneficial effects on the dystrophic heart both when acutely administered and in the long term. The mechanism(s) underlying these effects, however, have remained obscure.

We (Szabó et al., 2021) recently reported cardiac contractile dysfunction associated with disturbed intracellular Ca handling, classical features of the heart in DMD patients (Guan et al., 2014; Kamdar & Garry, 2016; Law et al., 2020; Sato et al., 2019), in a rat model of the human disease (DMD^mdx^ rats (Larcher et al., 2014)). In the present study, we used this animal model to test the acute effects of ivabradine administration on the dystrophic heart. We reasoned that ivabradine may rescue contractile dysfunction by improving intracellular Ca handling in dystrophic cardiomyocytes.

## 2 MATERIALS AND METHODS

### 2.1 Ethics statement

The investigation conformed to the guiding principles of the Declaration of Helsinki and coincided with the rules of the Animal Welfare Committee of the Medical University of Vienna. The experimental protocols were approved by the Austrian Science Ministry (ethics vote number: BMWFW‐66.009/0175‐WF/V/3b/2015).

### 2.2 Animal model

Male dystrophin‐deficient DMD^mdx^ Sprague Dawley rats (Larcher et al., 2014) originated from INSERM‐CRTI UMR 1064 (Nantes) and were bred at the Core facility for laboratory animal breeding and husbandry at the Medical University of Vienna. Genotyping of the rats was performed using standard PCR assays as described previously (Larcher et al., 2014).

### 2.3 Experimental protocol and transthoracic echocardiography assessment

Ten male DMD^mdx^ rats at the age of 9 months were anesthetized by intraperitoneal injection of a mixture of xylazine (4 mg/kg; Bayer) and ketamine (100 mg/kg; Dr E. Gräub AG), intubated (14‐gauge tube), and ventilated (9 mL/kg tidal volume, 75–85 strokes/min). Rectal temperature was measured and maintained at 37.5–38.5°C by a heated operating table. In five out of 10 rats, the right carotid artery was cannulated and a catheter inserted for measurement of mean arterial blood pressure (MABP) (PR‐409, 2F, Millar Instruments). Hemodynamic parameters were continuously recorded on a personal computer equipped with PowerLab System (8/30; both ADInstruments). In addition, the heart rate (HR) was determined from the electrocardiogram signal. The left jugular vein was cannulated for the administration of bolus injections of saline and ivabradine. After completion of the surgical preparation, the rats were allowed to stabilize for at least 15 min before they were treated with saline and ivabradine. First, saline was applied followed by ivabradine administration (iv; 1 mg/kg body weight). Serial transthoracic echocardiography assessment was performed prior (baseline) and 5 min after the saline, as well as 1 and 5 min after the drug administration, respectively. End‐diastolic (ED) and end‐systolic (ES) left ventricular (LV) diameters (D) were measured by M‐mode tracing. Left ventricular ejection fraction (LVEF) and fractional shortening (FS) were calculated using transthoracic echocardiograph ACUSON SC2000 Ultrasound System (Siemens Healthineers, 4V1c probe) as described previously (Pilz et al., 2019). Consistently, LVEDD, LVESD, and LVEF were determined by three independent measurements at midpapillary short‐axis view.

### 2.4 Isolation of ventricular cardiomyocytes

Additional four male DMD^mdx^ rats at the age of 9 months were anesthetized using isoflurane (2%, inhalation) and killed by cervical dislocation. Cardiomyocytes were isolated from the ventricles of their hearts using a Langendorff setup according to the myocyte isolation procedure described in detail in our previous work (Koenig et al., 2011). In brief, hearts were rapidly excised, and a cannula was inserted into the aorta for retrograde perfusion with Ca‐free solution containing 0.17 mg/mL Liberase TH (Roche) at 37°C for 18 min. The ventricles were then cut into pieces and incubated on a shaker at 37°C, and Ca concentration was increased to 200 μM over 1 h in five steps. Pieces of digested tissue were then triturated to liberate cardiomyocytes. After a centrifugation step, the cells were resuspended in Minimum Essential Medium (MEM) alpha (Gibco), containing ITS media supplement (Sigma) diluted 1:100, 4 mM l‐glutamine, 50 u/ml penicillin, 50 mg/mL streptomycin, and 25 mM blebbistatin (Sigma). Cells were finally plated on Matrigel (Becton Dickinson)‐coated culture dishes.

### 2.5 Intracellular Ca measurements

Ca transients were recorded from isolated ventricular cardiomyocytes at room temperature following the protocol described in our previous work (Rubi et al., 2018). In brief, myocytes preloaded with the cell membrane‐permeable Ca‐sensitive fluorescent dye Fluo‐4 am (Thermo Fisher Scientific) were bathed in an extracellular solution containing 140 mmoL/L NaCl, 4 mmoL/L KCl, 2 mmoL/L CaCl_2_, 2 mmoL/L MgCl_2_, 5 mmoL/L HEPES, 5 mmoL/L glucose, pH adjusted to 7.4 with NaOH. In order to elicit Ca transients, electrical stimulation was performed at 0.1 Hz via platinum electrodes in the bath. Dye fluorescence signals were acquired by means of a confocal microscope system (Nikon A1R+). Fluorescence peaks upon stimulation with single electrical pulses were analyzed relative to baseline fluorescence prior to stimulation (F0). For the measurements, the cardiomyocytes were bathed in extracellular solution in a 3,5 cm dish. Via an OctaFlow II perfusion system (ALA Scientific Instruments), myocytes in the detection window were first continuously superfused with extracellular solution (control). Thereafter, the cells were sequentially superfused with extracellular solution containing 0.1 μM and 1 μM ivabradine. Finally, the cells were superfused with extracellular solution again (washout). Always the average amplitude of the last three Ca transients prior to solution change (in steady‐state) yielded a single data point. Finally, to evaluate Ca transient duration, a single exponential function was fitted to the 10 s period of decaying fluorescence to obtain respective time constants (τ‐values). The use of a single exponential function for fitting revealed mean r^2^ values between 0.97 and 0.98 and was therefore considered appropriate.

### 2.6 Statistical analyses

Statistical analyses were performed with GraphPad Prism Software (version 7.03; GraphPad Software Inc.). Differences were evaluated by repeated measures ANOVA, followed by Tukey's multiple comparisons test, in case data showed a normal distribution. Friedman test, followed by Dunn's multiple comparisons test, was utilized in case the data were not normally distributed. A *p* value <0.05 was considered significantly different. Data were expressed as means ± SD.

## 3 RESULTS

**FIGURE 1:**
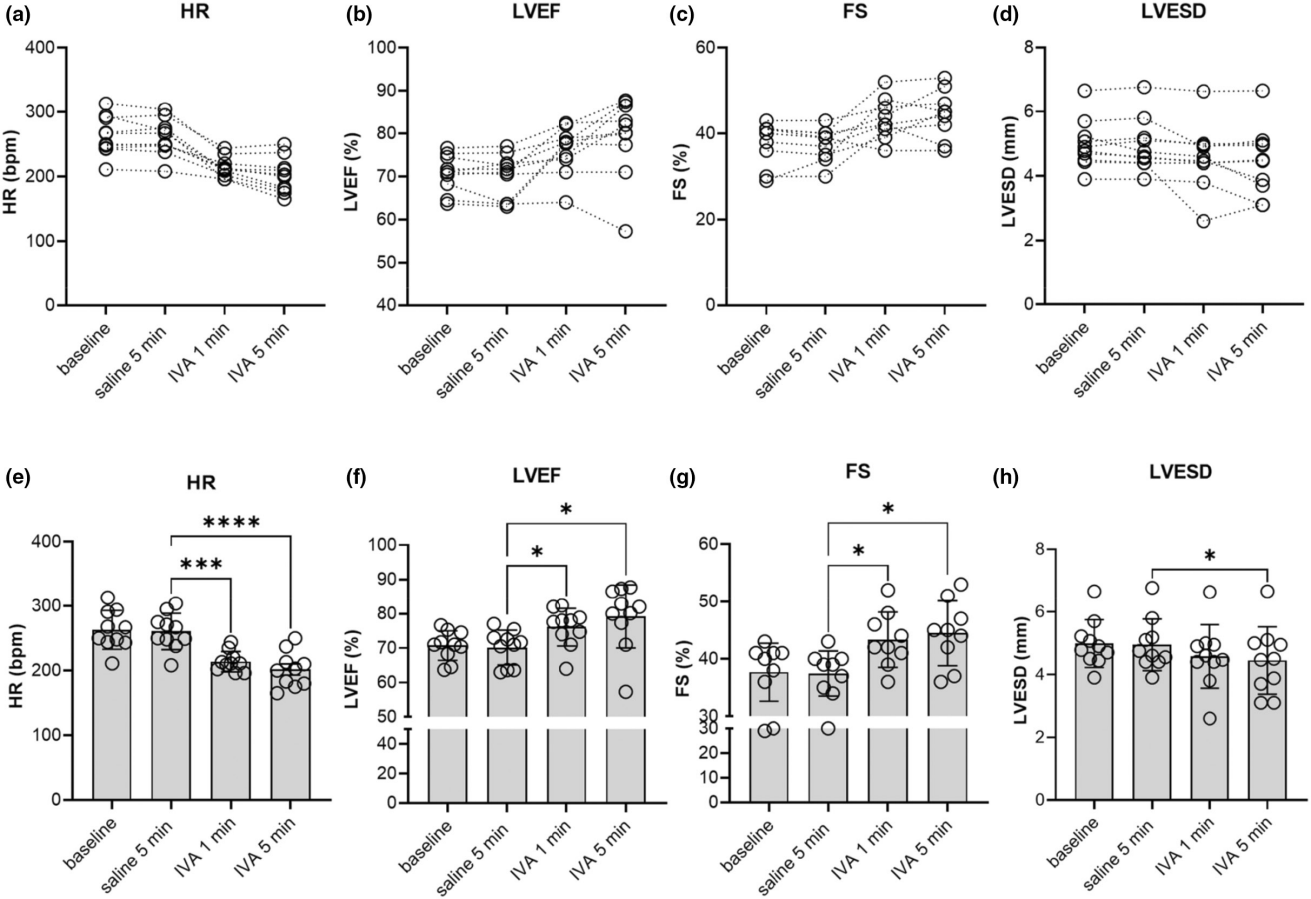
Acute effects of ivabradine (IVA) administration on cardiac contractile function in DMD^mdx^ rats in vivo. After saline administration, DMD^mdx^ rats were injected with 1 mg/kg body weight IVA (iv). Transthoracic echocardiography was performed on 2D M‐mode. (a–d) Changes in HR, LVEF, FS, and LVESD of individual rats at baseline, 5 min after saline injection, as well as 1 min and 5 min after the administration of IVA. Data points connected by dotted lines represent drug‐induced changes in HR (*n* = 10), LVEF (*n* = 10), FS (*n* = 10), and LVESD (*n* = 10). (e–h) Summarized echocardiographic parameters of left ventricular morphology and function derived from data displayed in a–d. (e) Effect of IVA on heart rate (HR). Data represent means ± SD. *p* < 0.001; repeated measures ANOVA. (f) Effect of IVA on left ventricular ejection fraction (LVEF); repeated measures ANOVA, *p* = 0.005. (g) Effect of IVA on fractional shortening (FS); repeated measures ANOVA, *p* = 0.01. (h) Effect of IVA on left ventricular end‐systolic diameter (LVESD); repeated measures ANOVA, *p* = 0.013. *p < 0.05; ***p < 0.001, and ****p < 0.0001 (Tukey's post hoc analysis).

Ivabradine's effects on cardiac function were studied in anesthetized DMD^mdx^ rats. The rats received an ivabradine dose of 1 mg/kg body weight via bolus iv injection. This dose of ivabradine in vivo resulted in a significant reduction in heart rate (HR) in comparison with saline (5 min after administration: approximately 20%; Figure 1a, e). This was comparable to the ivabradine‐induced HR reduction of about 15% observed in clinical settings (Fox et al., 2014). Simultaneously, we performed transthoracic echocardiography (*n* = 10) and recorded mean arterial blood pressure (MABP) (*n* = 5). Left ventricular ejection fraction (LVEF) significantly and rapidly increased by approximately 10–12% after the bolus injection of ivabradine (Figure 1b, f). Remarkably, the LVEF values in DMD^mdx^ rats in the presence of ivabradine were very similar to the basal LVEF values we previously reported for age‐matched healthy wild‐type rats (approximately 80%, (Szabó et al., 2021)). This suggests full restoration of cardiac functionality in the dystrophic heart by administration of the drug. Of note, two out of 10 rats did not show any improvement of LVEF despite a significant reduction in HR after ivabradine administration (see Figure 1b). Increased LVEF values were associated with a significant increase in fractional shortening (FS; Figure 1c, g) and a decrease in left ventricular end‐systolic diameter (LVESD; Figure 1d, h). Left ventricular end‐diastolic diameter (LVEDD) was not changed significantly by ivabradine administration (baseline: 7.7 mm vs. 5 min IVA treatment: 7.8 mm). Finally, ivabradine did not significantly change MABP values (Baseline: 80 mm Hg vs. 5 min IVA treatment: 78 mm Hg).

**FIGURE 2:**
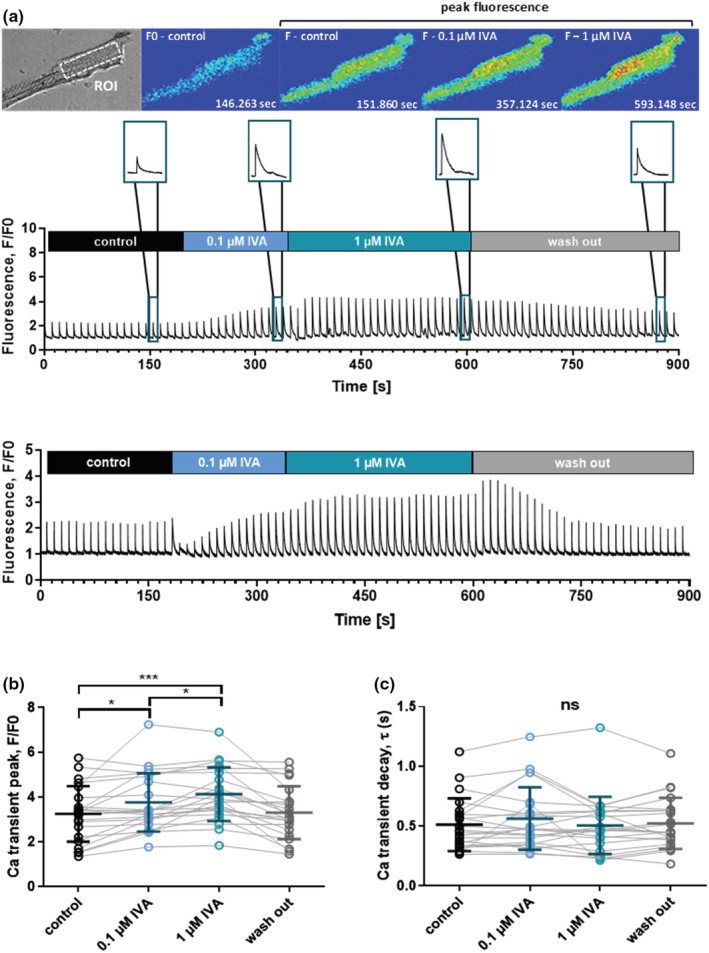
Acute effects of ivabradine (IVA) on intracellular Ca transients of ventricular cardiomyocytes derived from DMD^mdx^ rats. The Ca transients were electrically induced at a frequency of 0.1 Hz. (a) *Top panel*: Representative transmitted light image (top left), and image series of false color Fluo‐4 fluorescence during the course of a typical experiment as shown below. xyt image series was acquired with a sampling rate of 90 ms per frame, and emitted Fluo‐4 fluorescence was spatially averaged from a region of interest (ROI) drawn in the central region of a cardiomyocyte. *Middle and lower panel*: Two original trace examples of intracellular Ca measurements. The blue color in the bars indicates the time periods of external ivabradine application. The insets on top show representative single Ca transients at an enlarged time scale under control conditions, in the presence of 0.1 and 1 μM ivabradine, as well as after washout of the drug. (b) Comparison of steady‐state Ca transient amplitudes under control condition, and in the presence of two different concentrations of ivabradine, as well as after washout of the drug. The data points connected with solid lines originate from individual ventricular cardiomyocyte experiments, respectively. The cells (*n* = 22) were derived from four independent preparations (4 DMD^mdx^ rats). Data were also expressed as means ± SD. Repeated measures ANOVA revealed a significant difference between the experimental conditions (*p* < 0.001). *p < 0.05; ***p < 0.001 (Tukey's post hoc analysis). (c) Comparison of Ca transient decay kinetics under control conditions, and in the presence of two different ivabradine (IVA) concentrations, as well as after drug washout. The data points connected with solid lines originate from individual ventricular myocyte experiments, respectively. A single exponential function was fit to the decaying phase of the Ca transient in order to derive time constants (*τ*‐values). Ivabradine did not significantly affect Ca transient decay kinetics (ns, not significant; *p* = 0.33, Friedman test). Control experiments on ventricular cardiomyocytes isolated from a 9‐month‐old wild‐type Sprague Dawley rat revealed a significantly increased Ca transient peak (F/F0) in the presence of 1 μM ivabradine (3.6 ± 0.4 vs. 3.1 ± 0.4 in the absence of the drug; *n* = 11 cells; *p* < 0.01, Wilcoxon matched‐pairs signed rank test). Ca transient decay (*τ*‐value) was not affected by ivabradine (0.25 ± 0.06 s vs. 0.26 ± 0.04 s without drug; *p* = 0.83).

A potential explanation for the ivabradine‐induced enhancement of contractile performance in the heart of DMD^mdx^ rats is improved Ca handling in cardiomyocytes. To study this hypothesis, we tested the acute effect of the drug on electrically evoked intracellular Ca transients in isolated DMD^mdx^ ventricular cardiomyocytes. Figure 2 shows that superfusion of myocytes with bath solution containing low concentrations of ivabradine (100 nM and 1 μM) resulted in a significant increase in Ca transient amplitude. This suggested enhanced Ca release from the sarcoplasmic reticulum (SR) in the presence of ivabradine. Ivabradine's effect on Ca transient amplitude was typically permanent and lasted for the whole treatment duration (several min, e.g., see Figure 2a). In 7 out of 22 myocytes, however, ivabradine elicited only a temporary enhancement of Ca transient amplitude, which returned to control amplitude levels during superfusion with the drug. Currently, we do not have an explanation for the transient nature of the drug effect in some of the tested cells. Further of note, in a few other tested myocytes, ivabradine application failed to considerably increase Ca transient amplitude (Figure 2b), which matches with the lack of LVEF improvement in two ivabradine‐treated rats mentioned above. Permanent ivabradine‐induced enhancement of Ca transient amplitude (normal case) was fully reversible after washout of the drug (Figure 2b). In contrast to Ca transient amplitude, the decay phase of the transient was not affected by ivabradine (Figure 2c). This implied that, in DMD^mdx^ ventricular cardiomyocytes, ivabradine does not alter the speed of Ca removal from the cytosol after SR Ca release. Finally, control experiments on ventricular cardiomyocytes isolated from a 9‐month‐old wild‐type Sprague Dawley rat showed that ivabradine exerts similar effects on Ca transients as those observed in DMD^mdx^ myocytes (see legend of Figure 2).

## 4 DISCUSSION

Here, we show that administration of the negative chronotropic drug ivabradine to dystrophic DMD^mdx^ rats acutely improved cardiac function in this small animal model for human DMD. We also found that the amplitude of electrically induced intracellular Ca transients in ventricular cardiomyocytes isolated from DMD^mdx^ rats was increased by the drug. The latter effect, which occurred in vitro at drug concentrations comparable to those needed for HCN channel inhibition (see Introduction), suggested enhanced Ca release from the SR in the presence of ivabradine.

### 4.1 Ivabradine‐induced improvement of Ca handling in cardiomyocytes explains enhanced function in the dystrophic heart

Studies on animal models for DMD have revealed cardiac functional impairments and significant Ca handling abnormalities in dystrophic cardiomyocytes (Fauconnier et al., 2010; Gonzalez et al., 2014; Rubi et al., 2018; Szabó et al., 2021; Williams & Allen, 2007). Typically, electrically evoked intracellular Ca transients of dystrophic myocytes have decreased amplitudes (e.g., (Gonzalez et al., 2014; Szabó et al., 2021)), and their decay phase is significantly slowed (Rubi et al., 2018; Szabó et al., 2021; Williams & Allen, 2007), which suggests diminished SR Ca release and slowed removal of Ca from the cytosol, respectively. In the failing or injured mammalian heart, disturbed Ca handling in ventricular cardiomyocytes weakens contractile performance and, consequently, cardiac function (Benitah et al., 2003; Beuckelmann et al., 1992; Gambardella et al., 2018; Pieske et al., 1995). Particularly, diminished Ca release from the SR impairs cardiac systolic function, and slowed Ca removal from the cytosol after SR Ca release negatively affects diastolic function. These known facts regarding the failing heart, and the data of the current study, prompt us to propose that acutely administered ivabradine at least partly rescues systolic dysfunction in the dystrophic rat heart by increasing SR Ca release in cardiomyocytes. Enhanced systolic function was implied by significantly increased LVEF and FS. In addition, decreased LVESD in the presence of ivabradine was observed. The latter finding strongly suggests a moderate positive inotropic effect of the drug in the dystrophic heart.

The mechanism by which ivabradine increases electrically evoked Ca transients in dystrophic cardiomyocytes remains unknown. Xie et al. (2020) recently reported that the drug increases Ca transients in ventricular cardiomyocytes derived from healthy rats. This effect at the myocyte level was causally related to a positive ionotropic effect observed in rats following the administration of ivabradine. Because ivabradine also speeded the fast component of the Ca transient decay, drug‐induced increase in the Ca transient amplitude was ascribed to the activation of the sarcoplasmic‐endoplasmic reticulum Ca ATPase (SERCA) by the authors. We, however, can exclude this mechanism for dystrophic DMD^mdx^ rat cardiomyocytes, because ivabradine did not speed Ca transient decay after SR Ca release (Figure 2c). Also in control experiments with wild‐type rat cardiomyocytes, in our hands, ivabradine had no effect on Ca transient decay (see legend of Figure 2). As SERCA is the predominant mechanism for Ca removal from the cytoplasm, the Ca transient decay kinetics should have been speeded by ivabradine in case the drug would have augmented SERCA function. Alternatively, ivabradine could have increased electrically evoked Ca transients in dystrophic cardiomyocytes by activation of l‐type Ca channels. Thus, enhanced Ca influx through these channels could have augmented Ca‐induced Ca release from the SR. Our recently published findings (Haechl et al., 2019) showing that ivabradine up to 100 μM concentration did not affect l‐type Ca channel activity, however, precluded this possibility. Finally, factors directly or indirectly enhancing the activity of ryanodine receptors in the presence of ivabradine remain as potential mechanisms for drug‐induced enhancement of SR Ca release. With regard to ryanodine receptor activity, Xie et al. reported that ivabradine significantly enhanced SR Ca content and reduced Ca leak in cardiomyocytes from healthy rats (Xie et al., 2020). If this drug effect also occurs in dystrophic cardiomyocytes, enhanced Ca release via ryanodine receptors would be expected. Future studies are necessary to finally clarify ivabradine's mechanism(s) of action.

### 4.2 Potential effects of ivabradine on the dystrophic human heart

We recently reported that DMD^mdx^ rats show significantly impaired cardiac function consistent with dilated cardiomyopathy development (Szabó et al., 2021). In addition, intracellular Ca handling in ventricular cardiomyocytes derived from DMD^mdx^ rat hearts was significantly impaired. Cardiac dysfunction, development of a dilated cardiomyopathy (Kamdar & Garry, 2016; Law et al., 2020), and impaired intracellular Ca handling (Guan et al., 2014; Sato et al., 2019) are also features of the human dystrophic heart, and striking similarities between DMD^mdx^ rats and DMD patients are apparent. Thus, electrically evoked Ca transient amplitudes were reduced, and Ca transient decay kinetics was slowed both in DMD^mdx^ rat cardiomyocytes (Szabó et al., 2021) and in induced pluripotent stem cell‐derived cardiomyocytes from DMD patients (Guan et al., 2014; Sato et al., 2019). Similar Ca handling abnormalities were also present in cardiomyocytes isolated from the ventricular myocardium of patients with end‐stage heart failure caused by dilated cardiomyopathy (Beuckelmann et al., 1992; Beuckelmann et al., 1995). Together these findings suggest that the Ca handling abnormalities in the DMD^mdx^ rat heart closely mimic those observed in the heart of DMD patients and lead us to speculate that the ivabradine‐induced partial rescue of disturbed Ca handling we report herein for the DMD^mdx^ rat model may also work in the human dystrophic heart. In particular, enhancement of Ca release from the SR in cardiomyocytes in the presence of ivabradine may be beneficial for muscular dystrophy patients and improve cardiac contractility. This is in accordance with Finsterer et al., who reported that acutely administered ivabradine resolved heart failure in the case of a Becker muscular dystrophy patient, presumably by generating a positive inotropic effect (Finsterer et al., 2012).

### 4.3 Study limitations

The data of the present study were obtained by using the DMD^mdx^ rat model, and thus, we can only speculate that ivabradine exerts similar effects on the heart of DMD patients. Further, we have only tested acute effects of ivabradine at a single dosage in vivo, and at only two different concentrations in vitro. Finally, no attempts were made to elucidate the mechanism(s) underlying the observed effects, which would have been beyond the scope of a “Rapid report.”

We are currently studying the acute effects of ivabradine on the dystrophic heart in more detail. In addition, we are investigating the effects of chronic ivabradine treatment on cardiac function and cardiomyocyte Ca handling in DMD^mdx^ rats. Studies on various animal models for heart failure have suggested ivabradine‐mediated long‐term effects on Ca handling, which at least partially rescued disease‐associated cardiac functional abnormalities (Couvreur et al., 2010; Ma̧czewski & Mackiewicz, 2008; Navaratnarajah et al., 2013; Reil et al., 2013). Rescue of abnormal Ca handling in the dystrophic heart by ivabradine, thus, emerges as promising strategy for DMD pharmacotherapy.

## AUTHOR CONTRIBUTIONS

A.K. and K.H. conceived and designed research; P.L.S., J.M., and J.E. performed experiments; P.L.S., J.M., J.E., C.D., B.H., and J.S. analyzed data; P.L.S., J.M., B.K.P., H.K., H.T., X.K., A.K., B.H., and K.H. interpreted results of experiments; P.L.S., J.M., C.D., and J.S. prepared figures; A.K., K.H., H.T., and X.K. drafted the manuscript; J.E., B.K.P., H.K., H.T., and X.K. edited and revised the manuscript; all authors approved final version of the manuscript.

## FUNDING INFORMATION

This work was financially supported by “Österreichische Muskelforschung” (ÖMF) supported by the “Harley Davidson Charity Fonds” (AP00957OFF to K. Hilber and AP00935OFF to A. Kiss) and by the Austrian Science Fund ([FWF]; P30234‐B27 to K. Hilber).

## CONFLICT OF INTEREST STATEMENT

No conflicts of interest, financial or otherwise, are declared by the authors.
